# Emerging therapies for non-small cell lung cancer

**DOI:** 10.1186/s13045-019-0731-8

**Published:** 2019-04-25

**Authors:** Chao Zhang, Natasha B. Leighl, Yi-Long Wu, Wen-Zhao Zhong

**Affiliations:** 1 Guangdong Lung Cancer Institute, Guangdong Provincial Key Laboratory of Translational Medicine in Lung Cancer, Guangdong Provincial People’s Hospital and Guangdong Academy of Medical Sciences, Guangzhou, 510080 Guangdong People’s Republic of China; 2 Princess Margaret Cancer Centre, Toronto, Ontario Canada; 3 School of Medicine, South China University of Technology, Guangzhou, People’s Republic of China

**Keywords:** Tyrosine kinase inhibitors, Checkpoint inhibitors, CAR-T, Bispecific antibodies, Lung cancer

## Abstract

Recent advances in the field of novel anticancer agents prolong patients’ survival and show a promising future. Tyrosine kinase inhibitors and immunotherapy for lung cancer are the two major areas undergoing rapid development. Although increasing novel anticancer agents were innovated, how to translate and optimize these novel agents into clinical practice remains to be explored. Besides, toxicities and availability of these drugs in specific regions should also be considered during clinical determination. Herein, we summarize emerging agents including tyrosine kinase inhibitors, checkpoint inhibitors, and other potential immunotherapy such as chimeric antigen receptor T cell for non-small cell lung cancer attempting to provide insights and perspectives of the future in anticancer treatment.

## Background

In the past few decades, systemic treatment for lung cancer remained to be cytoxicity agents with platinum-based regimens. ECOG1594 was the first trial comparing four different chemotherapy regimens for advanced non-small cell lung cancer (NSCLC) head to head [1]. All chemotherapy regimens showed almost the same efficacy with objective response rate (ORR) of 19% and 7.9 m median overall survival (OS). The platinum-based doublet chemotherapy seemed to reach the plateau since then. In 2005, the first-ever trial combining small molecular targeted agent known as bevacizumab, an anti-vascular endothelial growth factor (VEGF) monoclonal antibody, with doublet chemotherapy, had shown superiority of overall survival with this treatment modality in advanced non-squamous non-small cell lung cancer patients without brain metastasis [2]. Yet, several trials including molecular targeted agents and chemotherapy fail to reach the endpoints [3–5].

Epidermal growth factor receptor, a well-known biomarker for targeted therapy at present, was first brought up with potential clinical responsiveness to tyrosine kinase inhibitor gefitinib in 2004[6]. Since then the era of targeted therapy was uncovered and multiple trials demonstrated the efficacy of tyrosine kinase inhibitor (TKI) in oncogene-driven non-small cell lung cancer patients [7–11]. In these trials, significantly improved progression-free survival (PFS) was observed compared to tradition chemotherapy; however, no overall survival benefit was identified which may be partly due to high crossover rate after disease progression [12–14]. Moreover, resistance to tyrosine kinase inhibitor was inevitable and sequential treatment was warranted [7, 15–17].

Although up to 69% of patients with advanced NSCLC could harbor actionable driver mutations, a number of patients barely got a chance for more effective agents other than chemotherapy [16, 18–21]. Until 2013, immunotherapy was crowned as the first place of scientific breakthroughs [22]. Efficacy of immunotherapy for those without targetable oncogene mutation was proven from second-line treatment [23–27] to first-line treatment [28, 29]. Through long-term follow-up, immunotherapy had also shown itself the greatest potential of long-term clinical benefit [30, 31], even though the efficacy was not that satisfactory [23–30]. Indeed, similar to targeted therapy, patients may eventually develop resistance to immunotherapy [32, 33] and some may even suffer hyperprogression after immunotherapy [34, 35]. The desire of novel agents that showed better efficacy, prolong survival benefit, and overcame resistance promoted the development of potential targets and corresponding drugs. In recent years, we have witnessed the birth of numerous emerging agents and their superior clinical responsiveness. Herein, we summarized the novel agents in tyrosine kinase inhibitors especially for epidermal growth factor receptor (EGFR) and anaplastic lymphoma kinase (ALK) inhibitors, checkpoint inhibitors, and other potential immunotherapy aiming to provide a landscape of emerging agents for NSCLC as well as insights and perspectives for the future in anticancer treatment.

### Epidermal growth factor receptor and human epidermal growth factor receptor 2 inhibitors

#### Dacomitinib

Dacomitinib is a selective and irreversible inhibitor for EGFR [36, 37]. In 2014, an official announcement from Pfizer indicated the trial failure of dacomitinib in patients with refractory advanced non-small cell lung cancer. However, based on superior results from phase II single-arm trial (ARCHER 1017) in the first-line setting, ARCHER1050, a phase III randomized control trial (ARCHER 1050) comparing dacomitinib and gefitinib head to head, was set to confirm its clinical efficacy and safety in expanded population. The results were promising, and the median PFS for dacomitinib and gefitinib was 14.7 months and 9.2 months, respectively (HR = 0.59, 95% CI 0.47–0.74) [38]. Similar efficacy was shown between EGFR 19Del and EGFR 21L858R which suggested opposite results compared to the first-generation TKI in previous researches [39, 40]. Further OS results have been recently unleashed, and the median OS was 34.1 m with dacomitinib versus 26.8 m with gefitinib (HR = 0.76, 95% CI 0.58–0.99) [41]. Higher incidence of adverse events compared to the first-generation TKI should be noticed [38, 41]. Currently, updated results of dose reduction in dacomitinib have been released and higher efficacy was found in dose modulation group [42]. Yet, efficacy results in patients with brain metastasis and resistant mechanism to dacomitinib were poorly explored and whether patients who had treatment failure after dacomitinib could still have a great chance of receiving osimertinib has not been answered [41]. Indeed, dacomitinib has been officially approved by the FDA in 2018 due to its superior performance in the first-line setting. Clinically, dacomitinib as a first-line treatment would be an optional choice, and hopefully, the third generation may be a salvage treatment after disease progression. But it would be too early to confirm its significant clinical role in first-line treatment neglecting the striking performance from osimertinib. Further clinical researches were warranted to provide evidence for a better therapeutic scheme.

#### Osimertinib (AZD9291)

**Fig. 1:**
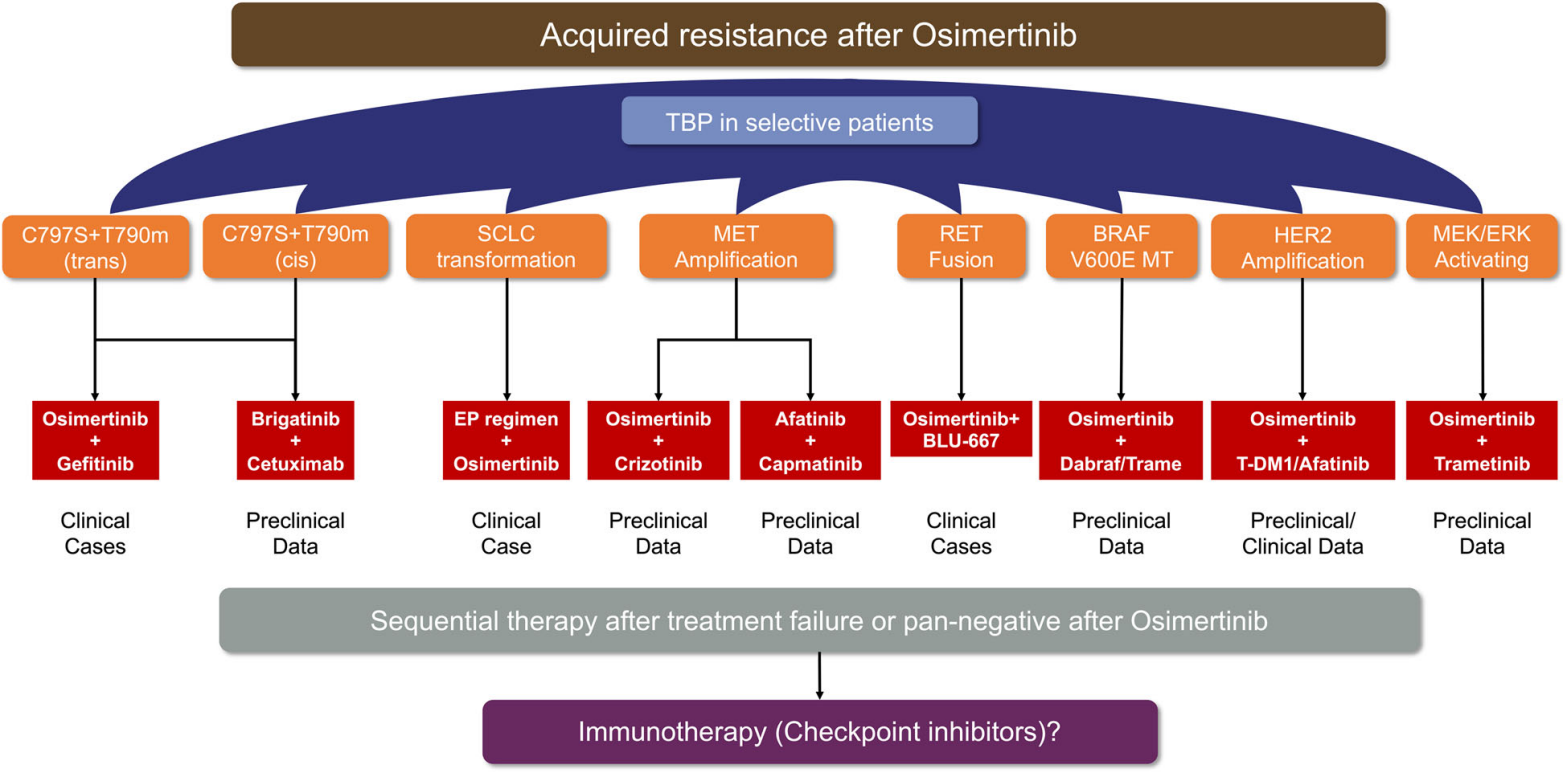
Reported acquired resistance to osimertinib and corresponding potential strategies. Preclinical and clinical data consisted of EGFR-dependent/independent resistant mechanism to osimertinib were included. Additional corresponding TKI with osimertinib may be available in other oncogene-driven resistance, and whether checkpoint inhibitors would be beneficial in pan-negative patients after osimertinib or treatment failure was yet to be answered. TBP, treatment beyond progression

Despite the high response rate to the first-generation TKI, majority of patients would suffer disease progression after 9–13 months of treatment [7, 15–17]. The most common resistant mechanism to the first-generation TKI is p.Thr790Met point mutation (T790 M) with almost 60% [15, 20]. Osimertinib, an irreversible third-generation TKI, was set to overcome resistance to T790M, and sensitive EGFR mutations (19Del and 21L858R) were covered as well [43, 44]. In a phase II single-arm AURA2 study [44], ORR was 70% (95% CI 64–77) among 199 pretreated patients receiving osimertinib and manageable side effect was identified. Extension population-based AURA study showed that 201 pretreated patients harboring T790 M mutation received osimertinib with a median treatment duration of 13.2 months. Objective response rate was 62% (95% CI, 54% to 68%), and median PFS was 12.3 months (95% CI, 9.5 to 13.8) [45]. Treatment-related adverse events were milder compared to previous TKI [7–10, 44, 45]. With the superior performance, the FDA has approved its indications in second-line treatment. To further demonstrate the efficacy of osimertinib in the first-line setting, FLAURA study has been put forward and preliminary results have been released. Osimertinib showed significantly prolonged PFS compared to standard EGFR-TKIs in first-line setting (18.9 months vs. 10.2 months, HR = 0.46, 95% CI 0.37–0.57) [46]. So far, the median overall survival for both osimertinib and standard EGFR-TKI group was not reached. Favorable trend for osimertinib could be identified with a *P* value of 0.007. Indeed, compared to previous EGFR-TKIs, osimertinib revealed much longer PFS and better efficacy as well as decreased toxicity. And since that, the FDA has approved its first-line setting in the early 2018. Yet, should the winner take it all? Recent studies have provided more evidence and faith of using osimertinib in the first line. The exploratory postprogression outcomes of phase III FLAURA study has been reported showing not reached median second PFS in osimertinib arm while 20 months for standard of care EGFR-TKI arm [47]. Another study found that the continuation of osimertinib after disease progression could lead to a median second PFS of 12.6 months and be associated with longer overall survival compared with discontinuation [48]. Indeed, the mature results of OS are requested to further give a final deposition of this issue. The other focal aspect for osimertinib is the resistant mechanism. Till now, limited studies reported the resistant mechanism of osimertinib and extreme complicated resistant profiles were identified based on current data [49–51]. Fortunately, several preclinical and small size studies have provided potential treatment modalities to overcome the resistance, but an umbrella trial should be designed to address the pending issues [48, 52–63]. On the other hand, considering the rapid development of checkpoint inhibitors (CPIs) in advanced NSCLC, whether CPIs could benefit in patients with pan-negative oncogenes after osimertinib or treatment failure of novel combination modality remained to be explored in prospective trials. (Figure 1). Among patients with advanced lung cancer, brain metastasis was regarded as one of the major factors for poorer prognosis [64–66]. In contrast to the first-generation EGFR-TKI, osimertinib showed much better response rate in brain metastasis which may be due to higher penetration through the blood-brain barrier (BBB) [67, 68]. Collectively, osimertinib would be a more competitive first-line treatment for advanced non-small cell lung cancer patients beyond the first-generation EGFR-TKIs and further OS data of FLAURA study was pending to decipher the order issue.

#### AZD3759

Over 50% of NSCLC patients with EGFR-activating mutations would develop CNS metastasis during treatment [65, 69, 70]. Poor survival was observed in these patients with 16 months for brain metastasis [64] and 4.5–11 months for leptomeningeal metastasis [66]. AZD3759 is an oral EGFR-TKI which was specifically designed to overcome the weak penetration of the blood-brain barrier [71, 72]. This drug contained no substrate for efflux transport [71] and achieved 100% penetration through BBB [69], suggesting superior clinical efficacy in CNS metastasis. The BLOOM study is a phase I, open-label, multicenter trial evaluating the safety and preliminary antitumor efficacy of AZD3759 [69]. Tolerable safety profile was observed in this trial, and high consistent concentration of AZD3759 between CSF and free plasma was observed. However, whether a high concentration of AZD3759 in CSF would be translated into durable CNS response and not inferior efficacy in extracranial target lesions compared to previous EGFR-TKI warrants further clinical results.

#### Poziotinib, TAK-788, afatinib, and pyrotinib

In NSCLC, approximately 10–15% of patients harbored EGFR-activating mutations. For those whose tumor has sensitive EGFR mutation including deletion in exon 19 and mutation encoding p.L858R, standard first-generation TKI could probably provide dramatic efficacy [7–11]. However, approximately 10–12% of patients within have an in-frame insertion in exon 20 of their tumors [73–75]. The EGFR exon 20 insertions are generally resistant to most EGFR-TKIs [76, 77] which may be due to the altered drug-binding pocket of exon 20 [76]. Poziotinib has been proven to be a potent inhibitor of both EGFR and HER2 exon 20 insertion mutations through preclinical models and clinical experience [78]. Its preliminary clinical activity has been reported in 2018 WCLC with confirmed ORR of 43% in advanced NSCLC [79]. Another novel agent for EGFR exon 20 insertion, TAK-788, has also been reported in 2018 WCLC [80]. The ORR was approximately 40% in NSCLC patients with EGFR exon 20 insertion. Remarkably, the disease control rate could reach up to 100% in this small group of population. Additionally, for HER2 mutations, afatinib has shown some activity through retrospective researches with limited prospective clinical trials assessing its efficacy in HER2 mutation patients [81–84]. A recent published phase II study (NICHE trial) showed a disease control rate of 53.8% with median PFS of 15.9 weeks and median OS of 56.0 weeks failing to verify the efficacy of afatinib in HER2 mutation patients [85]. On the other hand, pyrotinib is an oral, irreversible pan-HER receptor TKI. Preclinical data indicated effective antitumor activity in vitro and in vivo [86, 87]. A small sample size phase 2 study has investigated its efficacy and safety. Patients who received 400 mg of pyrotinib showed an ORR of 53.3% and a median PFS of 6.4 months regardless of prior treatment lines along with tolerable adverse events [88]. Indeed, further larger sample size evaluation was warranted to verify its clinical value.

### Anaplastic lymphoma kinase and ROS1 proto-oncogene receptor kinase inhibitors

#### Ceritinib

The current standard first-line treatment for advanced non-small cell lung cancer harboring ALK rearrangement is crizotinib [89]. Despite the rapid response to crizotinib, most of the patients would suffer disease progression within 12 months [90, 91]. Approximately, a resistant mechanism in one third of patients with ALK-rearranged NSCLC was owning to ALK-dependent mutation including tyrosine kinase domain or amplification of ALK fusion. Ceritinib is a small molecule, oral tyrosine kinase inhibitor of ALK [92]. In contrast to crizotinib, ceritinib is 20 times more potent as crizotinib for ALK fusion, yet no tumor activity against MET was observed. In a phase I study evaluating the antitumor activity and safety in NSCLC harboring ALK fusion, ceritinib showed 58% ORR in 114 patients with a dose of at least 400 mg. Median progression-free survival, although not that mature enough (38% patients censored), was 7.0 months with a median follow-up time of 9.5 months [93]. Overall adverse events rate of grade 3 or 4 related to ceritinib therapy was 49% (varied from 20–80% among different dose groups), majority of which was gastrointestinal (GI) issues. Based on these superior clinical outcomes, the FDA granted an accelerated approval to ceritinib for the treatment in NSCLC patients harboring ALK rearrangement [94]. In 2016, the updated results of ASCEND-1 was released. Two hundred fifty-five patients who received at least 1 dose of ceritinib 750 mg/day showed an overall response rate of 72% and 56% in treatment-naive and ALK inhibitor-pretreated groups, respectively. Intracranial disease control was reported in 79% of ALK inhibitor-naïve patients and 65% for ALK inhibitor-pretreated patients. However, approximately 81% of patients suffer at least 1 adverse events of grade 3 or 4 [95]. In a phase II trial (ASCEND-2), ceritinib was tested specifically in chemotherapy and ALK inhibitor-pretreated patients. ORR was 38.6% and median PFS was 5.7 months. The intracranial overall response rate was 45.0% with similar and manageable tolerability as previous researches [96]. Moving into the first-line setting, ASCEND-4, a phase III study, showed 72.5% ORR and median PFS of 16.6 months. Yet, the control arm of this study was chemotherapy without providing head to head comparison to crizotinib. Besides, adverse events of grade 3 or 4 (78.0%) were as high as previously reported with ceritinib 750 mg daily [97]. Due to that, ASCEND-8, a phase I study assessing the tolerability of different dose of ceritinib in ALK-positive NSCLC, was initiated [98]. Compared to 750 mg daily, 450 mg with food may be optimal with favorable gastrointestinal tolerability. Further updated analysis suggested consistent efficacy between 450 mg with food and 750 mg along with less GI toxicities [99].

Attributed to a similar molecular structure with ALK fusion, ROS1 fusion may also be potential beneficiaries with ALK-TKI [100]. For advanced NSCLC patients harboring ROS1 fusion, crizotinib was first reported to have an antitumor activity for the treatment of ROS1 fusion. The ORR was 72.0% with a median PFS of 19.2 months. Toxicities were mild with no treatment related to adverse events of grade 4 or 5 [101]. Results were further demonstrated in a larger East Asian population in a phase II study. To be noticed, 13.4% of ROS1-positive patients within the study received a complete response to crizotinib [102]. In a phase II single-arm study, the efficacy and safety of ceritinib were assessed in a small sample size population harboring ROS1 fusion [103]. ORR was 62% with a median PFS of 24 months in overall patients who received at least 2 prior systemic treatment. Grade 3 or 4 toxicities of ceritinib 750 mg daily in ROS1 fusion were much milder than in ALK fusion (37% vs. ~ 80%) which may probably be owing to the diverse interaction between drugs and targets.

#### Alectinib

Similar to EGFR mutation, patients with ALK rearrangement would be under high risk of brain metastasis [104]. Alectinib is a highly selective inhibitor of anaplastic lymphoma kinase (ALK) which has shown both systemic and central nerve system efficacy in ALK-positive non-small cell lung cancer [70, 105–108]. J-ALEX trial is the first trial comparing alectinib and crizotinib as the first-line setting in advanced non-small cell lung cancer with ALK rearrangement, but only involving the Japanese population [109]. The result of median PFS was rather promising with 20.3 months for alectinib and 10.3 months for crizotinib. The superiority had been duplicated in the upcoming ALEX trial involving a larger amount of population [110]. According to the updated results, the median PFS for alectinib in first-line treatment was 34.8 months which was almost three times longer than the standard first-line treatment for ALK rearrangement [89]. Besides, the phase III ALUR study directly compared alectinib with chemotherapy in crizotinib-pretreated ALK-positive non-small cell lung cancer [111]. Median PFS was 9.6 months with alectinib and 1.4 months with chemotherapy as second-line treatment indicating an absolute clinical role of alectinib as a first-line setting. Besides, in contrast to other ALK-TKI such as crizotinib, alectinib did not have a substrate for efflux transport [104, 112, 113] and penetration through BBB was significantly higher than crizotinib [114, 115]. Recent data revealed high objective response rate of 73.3% with 100% central nerve system (CNS) disease control rate (DCR) in patients with ALK rearrangement and symptomatic or large CNS metastasis [115]. Although the resistant profile of crizotinib has been well described [90, 116, 117], little was known about alectinib and Gly1202Arg (G1202R) remained a hot potato for alectinib [118, 119]. Indeed, considering its tremendous improvement in first-line treatment compared to other ALK TKIs, alectinib should currently be the first option from all aspects for treatment-naive patients with advanced non-small cell lung cancer harboring ALK rearrangement.

#### Brigatinib (AP26113)

Brigatinib, another second-generation highly potent ALK-TKI, was also designed for a broad range of ALK resistance mutations [120]. Similar to lorlatinib, brigatinib was proved to be efficiently inhibiting all clinically relevant ALK resistance mutations including ALK G1202R through preclinical models [121]. However, another study showed diverse outcome in preclinical models with IC50 of 129.5 nM to brigatinib indicating inferior sensitivity to G1202R [106]. In a multicenter retrospective study, one alectinib-pretreated patient harboring G1202R had a progressive disease as the best response to brigatinib [122]. Whether brigatinib could overcome the resistance to G1202R remained to be explored in a larger sample size trial. Through a phase II trial of brigatinib in patients with crizotinib-refractory ALK-positive NSCLC, brigatinib yielded both substantial systemic and intracranial response and 180 mg once daily was proven to have better efficacy with acceptable safety [123]. Currently, an interim analysis of ALTA-L1 has been reported, showing 51% of progression risk reduced although median PFS was not reached [124]. Although, relevant research suggested the brain accumulation of brigatinib may be restricted by P-glycoprotein (P-gp) and breast cancer resistance protein (BCRP) [125]. For patients with brain metastasis at baseline in ALTA trial, brigatinib achieved potent efficacy with a progression risk reduction of 73%. Similar adverse events were identified compared to previous ALK-TKI [89, 97, 126–128]. Most importantly, the final PFS and OS result of brigatinib should be expected to decipher whether brigatinib might be superior to the new standard first-line alectinib.

#### Lorlatinib

Lorlatinib is a highly potent and brain-penetrant third-generation ALK-TKI in patients with advanced ALK-positive NSCLC [106]. Most ALK-positive patients treated with first- or second-generation ALK-TKI would develop resistance to TKI including ALK Gly1202Arg (G1202R) solvent-front mutation located at the solvent-front region of ALK, and can impair drug binding through steric hindrance [106, 129]. A preclinical data showed lorlatinib was the only ALK inhibitor to potently inhibit wide-range ALK secondary mutations, including ALK G1202R [130]. So far for lorlatinib, only phase 1 and 2 study has been released, and the results were promising [131]. For treatment-naive patients, ORR was 90% and 69.5% for crizotinib-treated patients. Based on a preliminary analysis of paired cerebrospinal fluid and plasma samples, lorlatinib has been demonstrated with a high degree of penetration across the blood-brain barrier [104]. In this phase 1 and 2 trial, the intracranial response for treatment-naive patients was 66.7% while 87% for crizotinib-treated patients. Additionally, a phase I study evaluating the efficacy and safety of lorlatinib in ALK/ROS1-positive NSCLC showed encouraging results in either ALK rearrangement or ROS1 rearrangement patients regardless of treatment lines [132]. For ALK-positive patients, the overall ORR was 46% along with a median PFS of 9.6 months and 50% along with 7.0 months for ROS1-positive patients. Collectively, although PFS result was not that mature enough, considering its wide range profile for resistance to ALK-TKI, lorlatinib would be an optional sequential treatment for patients previously treated with ALK-TKI. A phase III study was being investigated comparing lorlatinib to crizotinib at first-line setting (NCT03052608), and the preliminary results may be presented in 2020.

#### Ensartinib (X-396)

Ensartinib (X-396) is a novel, aminopyridazine-based small molecule drug that could potently inhibit ALK. Through preclinical study, tenfold more potent than crizotinib inhibiting ALK-positive lung cancer cell lines was observed [133]. Results of a multicenter expansion study had been first reported in 2016 WCLC [134] showing similar response rate and adverse events compared to previous ALK-TKI. Currently, this first-in-human phase I/II multicenter study has revealed the survival benefit of ensartinib with median PFS of 26 months in treatment-naive patients and 9 months for crizotinib-pretreated patients [135]. ORR for treatment-naive patients was 80% and 69% for previously treated patients. Among patients with brain metastasis, intracranial disease control rate could reach up to 92.9%. eXalt3 is a phase 3 randomized trial comparing ensartinib and crizotinib head to head [136]. The preliminary results would be released in 2019 which would further verify and discuss its clinical role in ALK-positive lung cancer patients (Table 1)

**Table 1:** Overview of novel agents regarding TKI in advanced NSCLC including systemic and intracranial efficacy

Identifier	Trials	Agents	Phase	Indication	Population	ORR*	mPFS	mOS	iRR	imPFS/iDOR	Toxcicty (≧ grade3)
EGFR											
NCT00818441	ARCHER1070	Dacomitinib	II	First-line	Advanced lung cancer with clinical (never-smoker or former light smokers) or molecular selected	75.6%	18.2 months	40.2 months	NA	NA	NA
NCT01774721	ARCHER1050	Dacomitinib	III	First-line	Advanced NSCLC with one EGFR mutation (19Del or L858R)	75.0%	14.7 months	34.1 months	NA	NA	51%
NCT01802632	AURA2	Osimertinib	I/II	≧ Second-line	Advanced NSCLC progressed after at least one prior treatment involving EGFR-TKI	62.0%	12.3 months	NR	64.0%	7.1 months	NA
NCT02151981	AURA3	Osimertinib	III	Second-line	Advanced NSCLC presented with EGFR sensitive mutations and T790m after first-line EGFR-TKI	71.0%	10.1 months	NR	70.0%	8.9 months	NA
NCT02296125	FLAURA	Osimertinib	III	First-line	Untreated advanced NSCLC harbored sensitive EGFR mutations	80.0%	18.9 months	NR	76.0%	15.2 months	22%
NCT02228369	BLOOM	AZD3759	I	≧ Second-line	Advanced EGFR-mutant NSCLC with brain/leptomeningeal metastases progressed after EGFR-TKI and chemotherapy	65.0%	NR	NA	83.0%	NR	17% (200 mg), 40% (300 mg)
NCT03066206	/	Poziotinib	II	Unlimited	Advanced NSCLC bearing mutations/insertions of EGFR or HER2 exon 20 regardless of prior treatment	43.0%	5.6 months	NA	NA	NA	60%
ALK											
NCT01283516	ASCEND-1	Ceritinib	I	Unlimited	Locally advanced or metastatic cancer harboring genetic alterations in ALK (NSCLC)	72.0% (TN) 56.0% (PT)	18.4 months (TN) 6.9 months (PT)	NA	79.0% (TN)^#^ 65.0% (PT)^#^	8.2 months (TN) 11.1 months (PT)	81%
NCT01685060	ASCEND-2	Ceritinib	II	> Second-line	Advanced ALK-rearranged NSCLC with asymptomatic or neurologically stable baseline brain metastasis	38.6%	5.7 months	NA	45.0%	NA	71.4%
NCT01828099	ASCEND-4	Ceritinib	III	First-line	Untreated metastatic non-squamous NSCLC harboring ALK rearrangement	72.5%	16.6 months	NR	72.7%	16.6 months	78.0%
NCT01828112	ASCEND-5	Ceritinib	III	≧ Second-line	Advanced ALK-positive NSCLC with at least two prior systemic treatment including crizotinib	38.1%	5.4 months	18.1 months	35.0%	6.9 months	NA
NCT01801111	/	Alectinib	II	Second-line	Advanced NSCLC with ALK rearrangement after disease progression of prior crizotinib	50.0%	8.9 months	NR	57.0%	10.3 months	NA
JapicCTI-132316	J-ALEX	Alectinib (300 mg)	III	First-line	Advanced ALK-positive NSCLC with either chemotherapy naive or one previous chemotherapy	92.0%	25.9 months	NR	NA	NA	32%
NCT0207584	ALEX	Alectinib (600 mg)	III	First-line	Untreated advanced NSCLC harbored ALK rearrangment	82.9%	34.8 months	NR	81.0%	17.3 months	41%
NCT03052608	/	Loraltinib	I	Unlimited	Advanced NSCLC harbored ALK/ROS1 rearrangement regardless of prior treatments	46.0%	9.6 months	NA	42.0%	NA	NA
NCT01970865	/	Loraltinib	II	First-line	Untreated advanced NSCLC bearing ALK/ROS1-positive	90.0%	NR	NR	66.7%	NR	NA
				Second-line	Advanced ALK/ROS1 positive NSCLC with previous crizotinib	69.5%	NR	NR	87.0%	NR	NA
NCT01449461	/	Brigatinib	I/II	First-line	Treatment naive advanced ALK/EGFR positive NSCLC	100.0%	NR	NA	53.0%	15.6 months	36%
				Second-line	Advanced ALK/EGFR positive NSCLC with refractory disease after crizotinib	62.0%	14.5 months	NA	NA	NA	
NCT02737501	ATLA-L1	Brigatinib	III	First-line	Advanced ALK-positive NSCLC without previous ALK TKI treatment	71.0%	NR	NR	78.0%	NR	61%
ROS1											
NCT00585195	/	Crizotinib	I	Unlimited	Advanced NSCLC with a ROS1 rearrangement	72.0%	19.2 months	NR	NA	NA	NA
NCT01945021	/	Crizotinib	II	Unlimited	Advanced NSCLC with a ROS1 rearrangement	71.7%	15.9 months	32.5 months	73.9%	NA	25.2%
NCT00585195	/	Ceritinib	II	≧ Second-line	Advanced ROS1-positive NSCLC progressed after at least one prior systemic treatment	62.0%	9.3 months (CT) 19.3 months (CN)	24.0 months	25.0%^#^	NA	37.0%
NCT03052608	/	Lorlatinib	I	Unlimited	Advanced NSCLC harbored ALK/ROS1 rearrangement regardless of prior treatments	50.0%	7.0 months	NA	60.0%	NA	NA

*ORR* objective response rate, *mPFS* median progression-free survival, *mOS* median overall survival, *iRR* intracranial response rate, *imPFS* intracranial progression-free survival, *iDOR* intracranial duration of response, *TN* treatment naive, *PT* pretreated, *NR* not reach, *NA* not applicable

*ORR for EGFR-TKI was based on results regarding sensitive EGFR-activating mutations

^#^The disease control rate of intracranial response

.

### Other novel tyrosine kinase inhibitor

#### Entrectinib (RXDX-101), larotrectinib (LOXO-101), and LOXO-195

Recurrent gene fusions are one of the essential oncogenic drivers to promote tumor growth among varied malignancies [137]. Similar to ALK and ROS1 rearrangement, fusions of NTRK1, NTRK2, and NTRK3 are actionable drivers of tumor growth. The incidence of NTRK fusion in solid tumor was reported as 0.1% [138]. Entrectinib and larotrectinib were both inhibitors targeting NTRK fusions [139, 140]. Unlike larotrectinib, entrectinib also showed efficacy in ROS1 and ALK rearrangement [139]. Two phase I study (ALKA-372-001 and STARTRK-1) assessing entrectinib in NTRK-, ROS1-, and ALK-positive solid tumor showed promising efficacy and durable clinical benefit of 32 months in a ROS1-positive lung cancer patients [139]. Besides, promising intracranial efficacy was observed indicating high penetration through BBB.

Although limited data of larotrectinib was shown, a recent study containing three phase I/II clinical trials was published and splendid responsiveness was revealed in pan-solid tumor including lung cancer harboring NTRK fusion [140]. Responsiveness was observed regardless of tumor type, and the overall response rate was 80%. Based on its superb outcome, the FDA has approved its application in patients of solid malignancies harboring NTRK fusion. Despite the preliminary clinical results, primary and acquired resistance has already been characterized in several studies [141, 142]. To overcome the resistance mediated by acquired kinase domain mutations, LOXO-195, a selective TRK-TKI, was designed and preclinically proven to be highly potent in vitro [143]. Two patients whose tumor developed an acquired resistance to larotrectinib were treated with LOXO-195 and showed potential efficacy, but relevant data was warranted specifically in lung cancer patients for the future.

#### Repotrectinib (TPX-0005)

Similar to entrectinib and larotrectinib, repotrectinib is a next-generation TKI developed to inhibit clinically recalcitrant solvent front substitutions involving TRK, ROS1, and ALK. In a preclinical study, among common the acquired resistance to ALK, ROS1, and TRK including ALK G1202R, ROS1 G2032R, and TRKB G639R, repotrectinib showed high efficacy in vitro compared to other ALK/ROS1/TRK inhibitors. For patients with brain metastasis, a significant clinical challenge, this next-generation TKI showed superior efficacy compared to crizotinib in patients with CNS metastasis. This may be partly due to its smaller molecule structure compared to previous TKI drugs [144]. The current phase I/II clinical trial investigating the efficacy and safety of repotrectinib is still ongoing (NCT03093116), and further results should be expected.

#### RXDX-105, LOXO-292, and BLU-667

RET fusion is a well-established driver oncogene in a variety of malignancies. In lung cancer, RET fusion was found in 1–2% unselected cases [145]. RXDX-105 is an orally, VEGFR-sparing, multikinase inhibitor with activity against RET. Compared to other RET inhibitors including cabozantinib, vandetanib, and lenvatinib, RXDX-105 showed high preclinical activity [146–149]. In a phase I/Ib trial [150], treatment-naive NSCLC patients with RET fusion showed 19% ORR to RXDX-105 while 0% ORR for TKI-pretreated patients. Specifically looking into different upstream partners for RET, only non-KIF5B RET fusion showed satisfactory clinical efficacy to RXDX-105 which is similar to other RET inhibitors.

Unlike multikinase inhibitors such as RXDX-105 which may be under substantial “off-targets” hindering their clinical efficacy, LOXO-292 is a novel RET inhibitor with high selectivity [147, 149, 151, 152]. Through preclinical research and clinical experience with LOXO-292, it showed both high selectivity and responsiveness to RET fusion cell lines. Besides, LOXO-292 revealed high efficacy in KIF5B-RET fusion engineered cells which were different from previous RET inhibitors [151]. Another similar small molecule specifically targeting RET is BLU-667 which covered both RET fusion and RET-activating mutations as well [153]. Compared to RXDX-105, cabozantinib, and vandetanib, BLU-667 showed a broad range of efficacy in RET fusion and activating mutation with high selectivity in KIF5B-RET preclinically. A phase I, first-in-human study (NCT03037385) which tried to define the maximum tolerated dose and evaluate the safety along with antitumor activity is still ongoing, and clinical potentials of such high selective RET inhibitors will be elucidated in the future.

#### Capmatinib (INC280)

Capmatinib is a highly potent MET inhibitor [154], and its single-agent activity has been observed in preclinical models with strong MET amplification, overexpression, and mutations. MET amplification could be accounted for 5–26% in patients resistant to previous EGFR-TKI [20, 155–159]. Preclinical research suggested INC280 could restore sensitivity to erlotinib and promote apoptosis in EGFR-mutant NSCLC models [160]. As a clinical rationale for the combination of capmatinib and EGFR-TKI, a phase Ib/II single-arm trial evaluated the combination of INC280 and gefitinib in EGFR-TKI-pretreated patients [161]. Overall response rate across phase Ib/II regardless of MET copy number was 27%. In patients with high MET amplification (copy number ≥ 6), the ORR was 47% with acceptable adverse events. However, the survival data was not mature enough and the resistant mechanism of capmatinib has not been released yet. A study established MET-amplified NSCLC cell lines which showed an acquired resistance to capmantinib. With further examination, they found that the combined treatment of EGFR or PIK3CA would dramatically suppress cell proliferation and downstream signals [162]. This may partly suggest an alternative therapeutic strategy to overcome the resistance to capmatinib, but further clinical researches were required to elucidate.

#### Dabrafenib and trametinib

BRAF mutations occurred in about 2–4% of lung adenocarcinoma, and approximately 50% of them were BRAF V600E mutations [163, 164]. BRAF V600E mutations were reported to have shorter overall survival, and limited patients responded to chemotherapy compared to wild-type BRAF [165, 166]. Vemurafenib was the first BRAF V600E inhibitor assessed in a basket trial which indicated a 42% overall response rate within BRAF V600E mutation NSCLC [167]. Dabrafenib was a highly potent adenosine triphosphate-competitive inhibitor of BRAF kinase selective for the BRAF V600E mutations [168]. In a phase II non-randomized trial, the disease control rate (DCR) was 53% with a median PFS of 5.5 months. Serious adverse events were reported in 42% of patients [169]. Through preclinical study, dabrafenib plus trametinib had shown high antitumor activity in BRAF V600E mutation cell lines, and clinically, BRAF plus MEK inhibitors revealed improved clinical outcome in patients with BRAF V600E mutant metastatic melanoma [170, 171]. In two phase II non-randomized trials, consistent overall response rate was observed with 63.2% and 64.0% in previously treated and untreated patients, respectively. Similar median PFS was found as well in previously treated and untreated patients with 8.6 months and 10.9 months, respectively [172, 173]. Within previously treated patients, grades 3 and 4 events occurred in 49% of patients while almost 73% for untreated patients. So far, considering limited choices in BRAF mutation especially V600E mutations, dabrafenib plus trametinib should be the first option in these group of patients.

#### Anlotinib

Anlotinib is a novel, small molecule receptor tyrosine kinases (RTKs) and inhibits both tumor proliferation and angiogenesis [174–176]. Preclinical studies have shown that anlotinib has emerged much stronger anti-angiogenic activity than other anti-angiogenesis agents [177]. Clinically, the efficacy and safety of anlotinib was first demonstrated in a randomized phase II study as a third-line therapy in advanced NSCLC [178]. Patients in the anlotinib group showed a significantly longer PFS than the placebo group (4.8 months vs. 1.2 months). Although no statistical significance was shown in OS, favor trend of survival benefit was identified in the anlotinib group (9.3 months vs. 6.3 months). Final results of an expanded population phase III randomized trial (ALTER 0303) has been released last year in ASCO meeting and showed both prolonged PFS and OS in the anlotinib group with well-tolerable adverse events indicating anlotinib as a potential third-line treatment in advanced NSCLC patients.

### Checkpoint inhibitors

#### Pembrolizumab, nivolumab, and atezolizumab

With the rapid growth of immunotherapy these years, tradition chemotherapy in pan-negative advanced non-small cell lung cancer has been challenged from second-line treatment to first-line treatment by single-agent checkpoint inhibitors [23, 24, 27, 28]. For checkpoint inhibitors, the expression of PD-L1 has been considered as a major predictive factor for immunotherapy so far [179]. Given that significant discrepancy results of Keynote-024 and Checkmate-026[29, 180], only highly selective patients should be available for a single agent in the first-line setting. Back to the era of targeted therapy, combination strategies have achieved great success, and theoretically, this may probably work out in checkpoint inhibitors [181–183]. Keynote-021 first reported preliminary results of checkpoint inhibitors combined with chemotherapy [180]. Superior response rate and progression-free survival were observed with minor increased toxicities. Identical results were duplicated in phase III study Keynote-189 and Keynote-407 for lung adenocarcinoma and squamous cell carcinoma, respectively [184, 185]. To be noticed, the component of combination immunotherapy seemed to significantly influence the incidence of adverse events. Cisplatin or paclitaxel showed much better tolerance than carboplatin or nab-paclitaxel as combination components. In Keynote-042 (2018 ASCO meeting), single-agent pembrolizumab has broadened its indication to a larger population with PD-L1 positive. Yet, patients with high expression PD-L1 in both trials showed a similar clinical outcome. Considering the cost-effectiveness and toxicities, it would be optimal to provide single-agent pembrolizumab in patients with PD-L1 high expression while combination regimens for low or negative PD-L1 expression. As for nivolumab, even post hoc analysis with stratification of tumor mutation burden showed statistical significance, and it is still a negative trial showing no significant improvement between single-agent nivolumab and platinum-based chemotherapy in PD-L1-positive patients based on the study design probably due to high crossover rate and non-highly selective patients. Checkmate-227 was the first reported combination trial involving nivolumab in advanced NSCLC. Compared to standard platinum-based chemotherapy, nivolumab plus ipilimumab revealed a significantly improved ORR (45.3% vs. 26.9%) and prolonged mPFS (7.2 months vs. 5.5 months) in patients with high mutation burden regardless of PD-L1 expression [186]. Indeed, OS was not mature enough to present preliminary data and comparison between other arms as well as subgroup analysis was not released yet. Additionally, the three phase III trials of combination regimens involving atezolizumab (IMpower 150, IMpower131(2018 ASCO meeting), IMpower132(2018 WCLC meeting)) have all shown superiority in clinical outcome with tolerable adverse events in either non-squamous or squamous NSCLC [187]. Details of all posted trials with combination regimens are summarized in Table 2.

**Table 2:** Posted results of combination regimen trials for pembrolizumab, nivolumab, and atezolizumab in advanced non-small cell lung cancer

Identifier	Trials	Phase	Indication	Population	Intervention	mORR	mPFS	1-year PFS rate	mOS	1-year OS rate	Any cause of AE rates ≥grade 3
NCT02578680	Keynote-189	III	First-line	Treatment-naive metastatic non-squamous NSCLC without EGFR/ALK alteration	Platinum-based CT	18.9%	4.9 months	17.3%	11.3 months	49.4%	65.8%
					Platinum-based CT plus pembrolizumab	47.6%	8.8 months	34.1%	NR	69.2%	67.2%
NCT02775435	Keynote-407	III	First-line	Untreated metastatic squamous NSCLC without EGFR/ALK alteration	Carboplatin-based CT	38.4%	4.8 months	NA	11.3 months	48.3%	68.2%
					Carboplatin-based CT plus pembrolizumab	57.9%	6.4 months	NA	15.9 months	65.2%	69.8%
NCT02477826	Checkmate-227	III	First-line	Untreated recurrent NSCLC of high mutation burden without EGFR/ALK alteration	Platinum-based CT	26.9%	5.5 months	13.2%	NA	NA	36.0%*
					Nivolumab plus Ipilimumab	45.3%	7.2 months	42.6%	NA	NA	37.0%*
NCT02366143	IMpower150	III	First-line	Untreated metastatic non-squamous NSCLC (wild type group)	Bevacizumab plus carboplatin plus paclitaxel (BCP)	48.0%	6.8 months	18.0%	14.7 months^#^	60.6%^#^	50.0%^+^
					Atezolizumab plus BCP	63.5%	8.3 months	36.5%	19.2 months^#^	67.3%^#^	58.5%^+^
NCT02657434	IMpower131	III	First-line	Untreated metastatic squamous cell NSCLC without EGFR/ALK alteration	Carboplatin-based CT	41.0%	5.6 months	12.0%	13.9 months^#^	56.9%^#^	58.0%*
					Carboplatin-based CT plus atezolizumab	49.0%	6.3 months	24.7%	14.0 months^#^	55.6%^#^	69.0%*
NCT02657434	IMpower132	III	First-line	Untreated metastatic non-squamous NSCLC without EGFR/ALK alteration	Platinum-based CT	32.0%	5.2 months	17.0%	13.6 months^#^	55.4%^#^	59.0%
					Platinum-based CT plus atezolizumab	47.0%	7.6 months	33.7%	18.1 months^#^	59.6%^#^	69.0%

*CT* chemotherapy, *PFS* progression-free survival, *OS* overall survival, *ORR* objective response rate, *AE* adverse event

*Treatment-related adverse events

^+^Treatment-related adverse events (grade 3–4)

^#^Interim analysis results

#### Avelumab

Avelumab is a fully human immunoglobulin G1 (IgG1) monoclonal antibody [188]. Beyond pembrolizumab, nivolumab, and atezolizumab, it is one of the last PD-L1 inhibitors along with durvalumab to access the market. Avelumab had been first approved in the USA for the treatment of metastatic Merkel cell carcinoma. In contrary to other PD-1/PD-L1 drugs, the binding of avelumab to the surface of tumor cell via PD-L1 could induce natural killer cell-mediated antibody-dependent cellular cytotoxicity (ADCC) which may enhance its clinical efficacy [189, 190]. In a phase Ib, multicenter trial (JAVELIN Solid Tumor), patients with advanced, platinum-treated NSCLC were given a single-agent avelumab [188]. Acceptable safety profile was observed, and 50% of patients achieved disease control. Similar median progression-free survival and overall survival compared to previous PD-1/PD-L1 were observed with 17.6 weeks and 8.4 months, respectively [23, 26, 27]. Clinical efficacy was consistent with the level of PD-L1 expression, and higher expression of PD-L1 may be translated into a longer survival benefit. Recent results from a randomized phase 3 trial (JAVELIN Lung 200) also investigating the efficacy and safety of avelumab in platinum-treated patients with advanced NSCLC have been released [191]. In PD-L1-positive (≥ 1%) patients, no significant survival benefit was observed between the avelumab and docetaxel groups (11.4 months vs. 10.3 months) except the high PD-L1 expression groups (≥ 50% cutoff and ≥ 80% cutoff). Increased ORR was consistent with the higher expression of PD-L1 in avelumab group instead of docetaxel group indicating PD-L1 as an essential predictive biomarker for avelumab. However, according to the primary endpoint this trial set up initially, it is a negative study even with numerical significance in survival. Other relevant trials including JAVELIN Lung 100, JAVELIN Lung 101, and JAVELIN Medley were still ongoing (Table 3), and the closest report of JAVELIN Lung 100 will be released in 2019.

**Table 3:** Details of ongoing clinical trials for avelumab in early-stage and advanced-stage lung cancer

Objectives	Identifier	Title	Phase	Intervention	Study design	Population	Primary endpoint	Secondary endpoint	Status	Primary completion
Early-stage	NCT03050554	Phase I/II study of the safety, tolerability, and efficacy of stereotactic body radiation therapy (SBRT) combined with concurrent and adjuvant avelumab for definitive management of early stage non-small cell lung cancer (NSCLC)	I/II	Avelumab+SBRT	Single-arm trial	Stage I NSCLC with tumor(s) less than 5 cm in diameter or 250 cm^3^ in volume	Safety and tolerability, RFS	Locoregional control, OS	Recruiting	Oct 2020
Advanced-stage	NCT02576574	A Phase III, open-label, multicenter trial of avelumab (MSB0010718C) versus platinum-based doublet as a first line treatment of recurrent or stage IV PD-L1+non-small cell lung cancer	III	Avelumab	Randomized control trial	Metastatic or recurrent NSCLC without EGFR or ALK	PFS, OS	Best overall response, DOR, EQ-5D-5L	Active, not recruiting	2019 Oct
	NCT03472560	A phase II, open-label study to evaluate safety and clinical activity of avelumab in combination with axitinib in patients with advanced or metastatic previously treated non-small cell lung cancer or treatment naive cisplatin-ineligble urothelial cancer (JAVELIN MEDLEY VEGF)	II	Avelumab+axitinib	Single-arm trial	Pretreated advanced NSCLC with no more than 2 prior lines and EGFR/ALK/ROS1 negative	ORR	TTR, DOR, PFS	Recruiting	Sep 2020
	NCT03717155	A phase IIa, single-arm, multicenter study to investigate the clinical activity and safety of avelumab in combination with cetuximab plus gemcitabine and cisplatinin participants with advanced squamous non-small-cell lung cancer	II	Avelumab+cetuximab+gemcitabine+cisplatin	Single-arm trial	Advanced lung squamous carcinoma without EGFR mutation, ALK rearrangementand brain metastasis	Best overall response	Occurrence of treatment-emergent adverse events, PFS, DOR	Recruiting	Jan 2021
	NCT03568097	Phased avelumab combined with chemotherapy as first-line treatment for patients with advanced small-cell lung cancer (SCLC)	II	Avelumab+cisplatin (carboplatin)+etoposide	Single-arm trial	Treatment-naive advanced SCLC with untreated stable brain metastasis	1-year PFS	OS, best overall response, ORR	Recruiting	Nov 2020
	NCT02584634	A phase 1B/2, open-label, dose-finding study to evaluate safety, efficacy, pharmacokinetics and pharmacodynamics of avelumab in combination with either crizotinib or PF-06463922 in patients with advanced or metastatic non-small cell lung cancer	II	Group A: avelumab+crizotinib; group B: avelumab+PF-06463922	Non-randomized trial	Pretreated advanced NSCLC without ALK rearrangement for group A and unlimited prior lines with ALK positive for group B	DLT, overall response rate	DCR, OS	Active, not recruiting	Feb 2019
	NCT03268057	Phase 1b/study of VX15/2503 in combination with avelumab in advanced non-small cell lung cancer	I/II	VX15/2503+Avelumab	Single-arm trial	No prior immunotherapy treated NSCLC	DLT, AEs	ORR, DOR, PFS	Recruiting	May 2020
	NCT03317496	A multicenter, open-label, phase 1B/2 study to evaluate safety and efficacy of avelumab in combination with chemotherapy with or without other anti-cancer immunotherapies as first-line treatment in patients with advanced malignancies	II	Avelumab+pemetrexed/carboplatin	Non-randomized parallel trial	Untreated advanced non-squamous NSCLC without EGFR mutations or ALK rearrangement	DLT, ORR	PFS, DOR, TTR	Recruiting	Oct 2020
	NCT03158883	A pilot study of avelumab and stereotactic ablative radiotherapy in non-responding and progressing NSCLC patients previously treated with a PD-1 inhibitor	I	Avelumab+Stereotactic ablative radiotherapy	Non-randomized parallel trial	Immunotherapy pre-treated advanced NSCLC without EGFR mutations or ALK rearrangement	Overall response rate	OS, PFS, DCR	Recruiting	Jun 2020
	NCT02554812	A phase 1B/2, open-label study to evaluate safety, clinical activity, pharmacokinetics and pharmacodynamics of avelumab in combination with other cancer immunotherapies in patients with advanced maligancies	II	Avelumab+Utomilumab	Randomized parallel trial	Advanced NSCLC without EGFR mutation or ALK/ROS1 rearrangement regardless of prior lines treatment	DLT, ORR	TTR, DOR, PFS	Recruiting	Dec 2020

*PFS* progression-free survival, *OS* overall survival, *ORR* objective response rate, *DLT* dose-limiting toxicities, *DOR* duration of response, *TTR* time to response

#### Durvalumab

Durvalumab is a selective, high affinity human IgG1 monoclonal antibody which impedes PD-L1 from binding to PD-1 and CD 80 [192, 193]. Its clinical efficacy was first reported at the 2014 ASCO meeting through a phase I study, showing limited toxicity and potential response rate. On account of previous single-agent checkpoint inhibitor success in second-line treatment [23, 24, 26, 27], several phase trials of combination regimens involving durvalumab were initiated [194, 195]. Preliminary results of these studies showed no significant difference to other checkpoint inhibitors. One should notice that combination regimens involving durvalumab in both TATTON and CAURAL study revealed an extremely high risk of developing interstitial pneumonia, leading to the termination of both trials. The phase II ATLANTIC trial (NCT02087423) evaluated the efficacy of durvalumab as a third-line treatment in advanced NSCLC [196]. The ORR was 7.5%, 16.4%, and 30.9% in patients with PD-L1 expression of < 25%, > 25% and > 90%, respectively. PFS in patients with high PD-L1 and low/negative PD-L1 expression was 3.3 and 1.9 month. To be noticed, cohort 1 in this trial receiving single-agent durvalumab included advanced NSCLC patients with EGFR-sensitive mutations or ALK rearrangement. The ORR in this group was not remarkable even in PD-L1 high expression population. Two well-known phase III trials with diverse ending were unleashed last year. The MYSTIC trial (NCT02453282) assessing durvalumab plus tremelimumab or durvalumab monotherapy versus platinum-based chemotherapy showed both combination and single-agent regimen which failed to reach the primary endpoint without PFS benefit. The PACIFIC trial (NCT02125461), on the other hand, achieved great success and led to a treatment paradigm shift for unresectable locally advanced NSCLC [197, 198]. Additionally, several phase III trials of durvalumab are pending or ongoing, and hopefully, more optional treatment would be provided (Table 4).

**Table 4:** Ongoing phase III trials for durvalumab in early-stage and advanced-stage lung cancer

Objectives	Identifier	Title	Intervention	Study design	Population	Primary endpoint	Secondary endpoint	Status	Primary completion
Early-stage	NCT03703297	A phase III, randomized, double-blind, placebo-controlled, multi-center, international study of durvalumab or durvalumab and tremelimumab as consolidation treatment for patients with stagei-iii limited disease small-cell lung cancer who have not progressed following concurrent chemoradiation therapy (ADRIATIC)	• Durvalumab+placebo • Durvalumab tremelimuab, placebo+placebo	Randomized parallel trial	Limited-stage SCLC without progression after definitive concurrent chemoradiation	PFS, OS	ORR, TTD/TTM, PFS2	Recruiting	Jun 2021
	NCT03800134	A phase III, double-blind, placebo-controlled, multi-center international study of neoadjuvant/adjuvant durvalumab for the treatment of patients with resectable stages II and III non-small cell lung cancer (AEGEAN)	• Durvalumab+platinum-based chemotherapy • Placebo+platinum-based chemotherapy	Randomized parallel trial	Resectable stage IIA-IIIB NSCLC	MPR	pCR, OS, DFS	Recruiting	Jul 2020
	NCT03519971	A phase III, randomized, placebo-controlled, double-blind, multi-center, international study of durvalumab given concurrently with platinum-based chemoradiation therapy in patients with locally advanced, unresectable non-small cell lung cancer (StageIII) (PACIFIC2)	• Durvalumab+platinum-based chemotherapy and radiation • Placebo+platinum-based chemotherapy and radiation	Randomized parallel trial	Unresectable locally advanced stage III NSCLC	PFS, ORR	OS, DOR, PFS2	Recruiting	Sep 2020
	NCT02273375	A phase III prospective double blind placebo controlled randomized study of adjuvant MEDI4736 in completely resected non-small cell lung cancer	• Durvalumab • Placebo	Randomized parallel trial	Stage IB (> 4 cm) to IIIA NSCLC after complete surgical resection	DFS	OS, LCSS	Recruiting	Jan 2023
	NCT03706690	A phase III, randomized,double-blind,placebo-controlled,study of durvalumab as consolidation therapy in patients with locally advanced, unresectable NSCLC, who have not progressed following definitive, platinum-based chemoradiation therapy (PACIFIC5)	• Durvalumab • Placebo	Randomized parallel trial	Unresectable locally advanced stage III NSCLC	PFS	OS, ORR, DOR	Recruiting	Mar 2021
Advanced-stage	NCT03164616	A phase III, randomized, multi-center, open-label, comparative global study to determine the efficacy of durvalumab or durvalumab and tremelimumab in combination with platinum-based chemotherapy for first-line treatment in patients with metastatic non small-cell lung cancer (NSCLC) (POSEIDON)	• Durvalumab+tremelimumab • Durvalumab monotherapy+SoC • SoC chemotherapy alone	Randomized parallel trial	Untreated advanced NSCLC without activating EGFR mutation or ALK fusions	PFS, OS	ORR, DOR, PFS2	Recruiting	Sep 2019
	NCT03003962	A phase III randomized, open-label, multi-center study of durvalumab (MEDI4736) versus standard of care (SoC) platinum-based chemotherapy as first line treatment in patients with PD-L1-high expression advanced non small-cell lung cancer	• Durvalumab • SoC chemotherapy	Randomized parallel trial	Untreated advanced PD-L1 positive NSCLC without EGFR mutation and ALK rearrangement	OS	ORR, DOR, PFS	Recruiting	Sep 2019
	NCT02453282	A phase III randomized, open-label, multi-center, global study of MEDI4736 in combination with tremelimumab therapy or MEDI4736 monotherapy versus standard of careplatinum-based chemotherapy in first line treatment of patients with advanced or metastatic non small-cell lung cancer (MYSTIC)	• Durvalumab • Durvalumab+tremelimumab • SoC chemotherapy	Randomized parallel trial	Untreated advanced NSCLC without activating EGFR mutation or ALK fusions	OS, PFS	ORR	Active, not recruiting	Oct 2018
	NCT03043872	A phase III, randomized, multicenter,open-label, comparative study to determine the efficacy of durvalumab or durvalumab and tremelimumab in combination with platinum-based chemotherapy for the first-line treatment in patients with extensive disease small-cell lung cancer (SCLC) (CASPIAN)	• Durvalumab+tremelimumab+EP • Durvalumab+EP • EP	Randomized parallel trial	Untreated extensive stage SCLC	OS, PFS	ORR, EORTC QLQ-C30	Active, not recruiting	Sep 2019
	NCT02542293	A phase III randomized, open-label, multi-center, global study of medi4736 in combination with tremelimumab therapy versus standard of care platinum-based chemotherapy in first-line treatment of patients with advanced or metastatic non small-cell lung cancer (NSCLC)	• Durvalumab+tremelimumab • SoC chemotherapy	Randomized parallel trial	Untreated advanced NSCLC without activating EGFR mutation or ALK fusions	OS	PFS, ORR, DOR	Active, not recruiting	Mar 2019
	NCT02352948	A phase III, open label, randomized, multi-centre, international study of MEDI4736, given as monotherapy or in combination with tremelimumab determinedby PD-L1 expression versus standard of care in patients with locally advanced or metastatic non-small cell lung cancer (stage IIIB-IV) who have received at least two prior systemic treatment regimens including one platinum based chemotherapy regimen and do not have known EGFR activating mutations or ALK rearrangements (ARCTIC)	• Durvalumab • Durvalumab+tremelimumab • Tremelimumab • Vinorelbine/gemcitabine/erlotinib	Randomized parallel trial	Advanced NSCLC without EGFR mutations and ALK rearrangment after progression of chemotherapy and at least one prior regimens treatment	OS, PFS	ORR, DOR	Active, not recruiting	Feb 2018

*DCR* disease control rate, *LCSS* lung cancer-specific survival, *PFS2* time from randomization to second progression, *TTD/TTM* time to death/time to distant metastasis, *MPR* major pathological response, *pCR* pathological complete response, *DFS* disease-free survival

### Potential novel treatment modalities for lung cancer

#### Chimeric antigen receptor T cell and bispecific antibodies

Beyond the field of checkpoint inhibitors, another immunotherapy such as adoptive cellular immunotherapy has emerged as a remarkable treatment modality in the past decades [199, 200]. Unlike checkpoint inhibitors, which induce antitumor activity through blocking the barrier between effective T cells and tumor cells [201–203], adoptive cellular immunotherapy is a novel approach providing “artificial” effective T cells to specifically target tumor cells directly regardless of tumor types [204–206]. With that being said, adoptive cellular immunotherapy may bring broad range effect on different tumors compared to checkpoint inhibitors, which have been reported diverse response among a variety of tumors [207–213]. So far, significant advances in chimeric antigen receptor T cell (CAR-T) have been accelerated in hematological malignancies especially for CD19-targeted CAR-T-cell therapy in leukemia [214–218]. However, in solid tumors, it is tough to design CAR-T because no such surface antigen as unique as CD19 has yet been identified [219]. Indeed, numerous clinical trials of CAR-T regarding lung cancer have been initiated including tumor-associated antigen (TAA) of EGFR (NCT02862028, NCT01869166), HER2 (NCT00889954, NCT01935843), carcinoembryonic antigen (CEA) (NCT01723306, NCT02349724), and mesothelin (MSLN) (NCT01583686, NCT03054298). Gladly, several trials focusing on NSCLC were initiated and ongoing in China [220]. One clinical trial of EGFR-specific CAR-T regarding non-small cell lung cancer (NCT01869166) had reported its preliminary results. 45.5% (5/11) of advanced NSCLC patients achieved stable disease, and 2 achieved partial response (PR). Treatment-related adverse events were manageable indicating its potentials in NSCLC. Yet, several aspects regarding the application of CAR-T in solid tumors should be noticed. First, the “off-target” effect is one of the major causes that lead to increased toxicity and less efficacy. In hematological malignancies, for example, the B cell acute lymphoblastic leukemia (B-ALL), well tolerance could be observed during the treatment of CD19-targeted CAR-T cells [221] due to the ubiquitous expression of CD19 on differentiated B cells instead of hematopoietic stem cells. On the contrary, target antigen in a solid tumor may be additionally expressed in other tissue or organs which may lead to unexpected treatment-related toxicity. Besides, it is much more difficult to select candidate antigen in solid tumors with higher antigen heterogeneity [222–224]. Second, microenvironment in solid tumors was relatively immunosuppressive-preferred compared to hematological malignancies leading to less efficient CAR-T therapy in solid tumors. Indeed, ongoing trials regarding solid tumors may further decipher the uncertainty in the future.

The concept of bispecific antibody for oncogene was based on the simultaneous activation of different pathways driving tumor proliferation and growth [225–227]. So far, the bispecific antibody for lung cancer is still under initial researches. It is indeed an encouraging agent for lung cancer in the future according to the preliminary results. The novel EGFR/cMet bispecific antibody (JNJ-61186372), a fully humanized IgG1 antibody, was first put up in 2016, and its preliminary results in human were reported in 2018 WCLC [228, 229]. Objective response was shown in various activating EGFR mutations including T790m and exon 20 insertion. It seemed like the two separate targets combined may broaden its antitumor activity compared to single-target inhibition. However, the efficacy and duration of response along with adverse events are warranted to further clarify its clinical application.

## Discussion

**Fig. 2:**
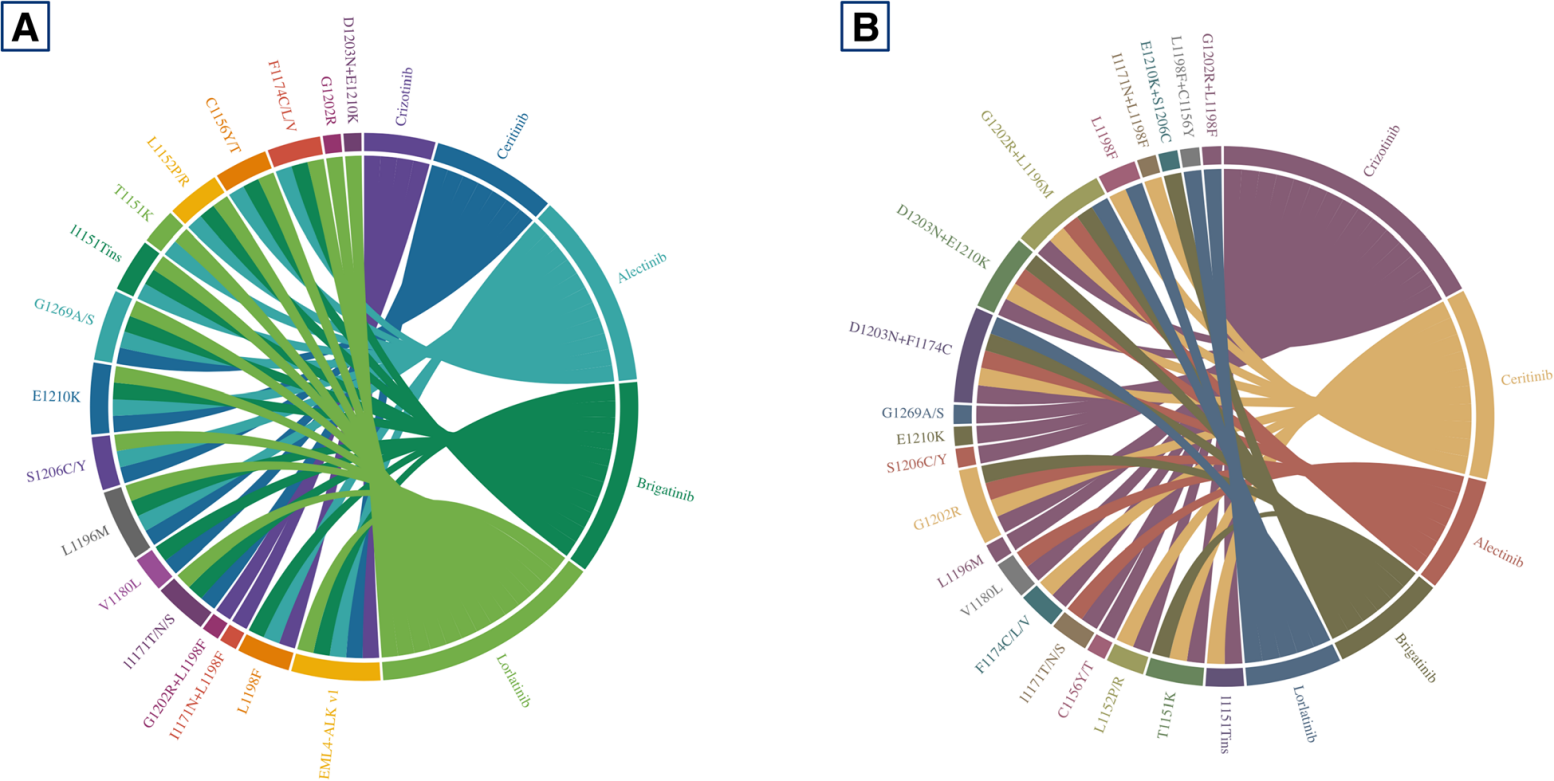
Chord diagrams for sensitive and resistant mutations regarding ALK-TKIs. Both preclinical data and clinical reported cases (preferred) were enrolled to determine the efficacy of ALK-TKIs to different ALK-dependent mutations. Crizotinib had smallest sensitive mutation profiles compared to lorlatinib while opposite in resistant profiles. **a** Mutation profiles showed responsiveness to different ALK-TKIs. **b** Mutation profiles reported to be resistant to different ALK-TKIs

**Fig. 3:**
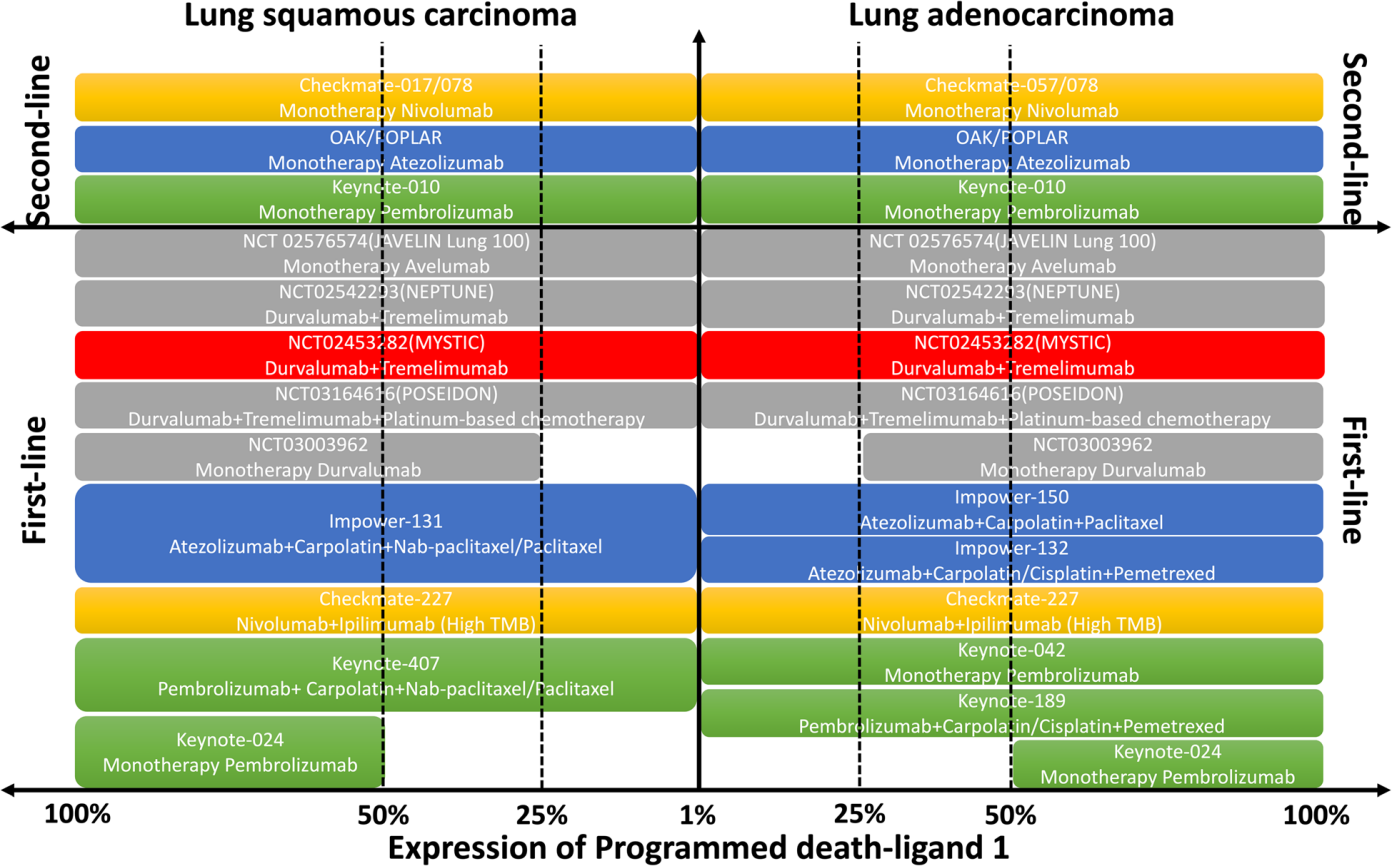
Results of posted and pending trials of PD-1/PD-L1 inhibitors between lung adenocarcinoma and squamous carcinoma regarding different PD-L1 expression. All posted and pending trials were stratified based on the indication for different expression of PD-L1 and treatment lines. Only PD-1/PD-L1 inhibitors of pembrolizumab, nivolumab, atezolizumab, durvalumab, and avelumab were included except for checkpoint inhibitors from Chinese pharmaceutical companies due to early phase trials of these checkpoint inhibitors for lung cancer. Checkmate-227, although regardless of PD-L1 expression, required high tumor mutation burden (TMB)

**Fig. 4:**
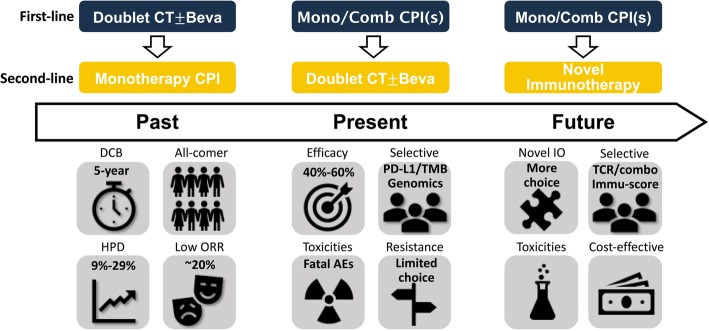
Perspectives for the evolving modalities of immunotherapy in NSCLC. Treatment modalities involving immunotherapy in NSCLC had evolved from second-line setting to first-line setting. Prior immunotherapy, highly selective patients, and combination strategies had raised significant efficacy improvement but increased toxicities as well. Novel immunotherapy in the future combined with multiple novel biomarkers may infinitely consolidate the clinical role of immunotherapy in advanced NSCLC

Novel agents for lung cancer have been booming these years. Since the post era of IPASS and 2013 when immunotherapy has been crowned as one of the breakthroughs, remarkable clinical results from both tyrosine kinase inhibitors and checkpoint inhibitors for lung cancer have generated a great number of potential agents which significantly improved patients’ survival beyond the era of chemotherapy. The current advances have been undoubtedly shifting the clinical paradigm for advanced lung cancer. So far, numerous potential agents including TKIs, CPIs, and underlying treatment modalities other than what we have mentioned above are under preclinical researches or early phase trials. Compared to previous standard treatment regarding TKIs, novel agents showed significant improvement in several aspects including improvement of BBB penetration, broadened target profiles, overcoming resistant mechanism, prolonged survival, and lower toxicity. In EGFR-TKIs, osimertinib has been undoubtedly claiming its essential clinical role in the first-line setting. However, whether sequential treatment following combination modalities (NEJ009 and JO25567 presented in 2018 ASCO meeting) or even novel second-generation TKI dacomitinib would be translated into better clinical outcome remained unknown. With potentially manageable acquired resistance, osimertinib would be temporally the best first option for untreated EGFR-mutant NSCLC patients. For ALK rearrangement, on the other hand, numerous highly potent targeted drugs were innovated and approved these years. Extremely complex mutational profiles were observed after the treatment of ALK-TKIs. Through chord diagram (Fig. 2) regarding sensitive and resistant mutational profiles of corresponding ALK-TKIs integrated from previous researches [106, 230–242], it is easy to tell that novel ALK-TKIs could cover more ALK-dependent mutations other than ALK fusion and meanwhile show antitumor activity in more resistant subtypes. Concurrent ALK-resistant mutations remained to be unyielding fields at the moment, and combination of different ALK-TKIs might be worthy to try in the future. For CPIs, numerous similar checkpoint inhibitors targeting PD-1/PD-L1 including CPIs developed by Chinese pharmaceutical companies [243] have been generated since the heat of immunotherapy. The clinical paradigm for wild-type NSCLC has been undoubtedly shifted with so many approved single or combination regimens involving CPIs in the first- and second-line setting. Some other phase III trials of novel checkpoint inhibitors were pending, and hopefully, more choices would be available in the first-line setting (Fig. 3). Indeed, due to first-mover advantage and limited understanding of how immunotherapy works within microenvironment, novel CPIs would be much tougher to compete as a single agent with previous CPIs. Combination treatment modality and clinical unmet needs, being the two major aspects, were ideal resolutions for the development of novel CPIs. To be noticed, challenges to immunotherapy remained to be unsolved including hyperprogression [244, 245], immune-related toxicities [246, 247], and primary/adaptive resistance to immunotherapy [248]. Moreover, potential novel treatment modalities have aroused great interest and their preliminary performances revealed remarkable prospects for development in lung cancer. Yet, most of the novel agents were still under the early stage of birth and further results should be expected. Whether these novel immunotherapy modalities may take place after the treatment failure of first-line checkpoint inhibitors in the future would be worth looking forward to (Fig. 4). At this moment, we are facing the condition of numerous novel agents developed for lung cancer. Improvement of clinical trials accelerating the application of novel drugs in clinical practice and discovery of novel effective targets along with much more precise biomarkers would be so much essential for anticancer treatment in the future.

## Abbreviations

ADCC: Antibody-dependent cellular cytotoxicity
ALK: Anaplastic lymphoma kinase
B-ALL: B cell acute lymphoblastic leukemia
BBB: Blood-brain barrier
CAR-T: Chimeric antigen receptor T cell
CEA: Carcinoembryonic antigen
CNS: Central nerve system
CPI: Checkpoint inhibitor
DCR: Disease control rate
EGFR: Epidermal growth factor receptor
IgG1: Immunoglobulin G1
MSLN: Mesothelin
NSCLC: Non-small cell lung cancer
ORR: Objective response rate
OS: Overall survival
PD-1/PD-L1: Programmed cell death protein-1/programmed cell death protein ligand-1
PFS: Progression-free survival
PR: Partial response
RTK: Receptor tyrosine kinases
TAA: Tumor-associated antigen
TKI: Tyrosine kinase inhibitor
